# One-Step Microwave-Assisted Synthesis of PtNiCo/rGO Electrocatalysts with High Electrochemical Performance for Direct Methanol Fuel Cells

**DOI:** 10.3390/nano11092206

**Published:** 2021-08-27

**Authors:** Kun-Yauh Shih, Jia-Jun Wei, Ming-Chi Tsai

**Affiliations:** Department of Applied Chemistry, National Pingtung University, Pingtung County 90003, Taiwan; sg974408@gmail.com (J.-J.W.); mikechi614@gmail.com (M.-C.T.)

**Keywords:** direct methanol fuel cells, PtNiCo/rGO, graphene, microwave-assisted synthesis, nanocomposites

## Abstract

Platinum (Pt) is widely used as an activator in direct methanol fuel cells (DMFCs). However, the development of Pt catalyst is hindered due to its high cost and CO poisoning. A multi-metallic catalyst is a promising catalyst for fuel cells. We develop a simple and rapid method to synthesize PtNiCo/rGO nanocomposites (NCs). The PtNiCo/rGO NCs catalyst was obtained by microwave-assisted synthesis of graphene oxide (GO) with Pt, Ni, and Co precursors in ethylene glycol (EG) solution after heating for 20 min. The Pt-Ni-Co nanoparticles showed a narrow particle size distribution and were uniformly dispersed on the reduced graphene oxide without agglomeration. Compared with PtNiCo catalyst, PtNiCo/rGO NCs have superior electrocatalytic properties, including a large electrochemical active surface area (ECSA), the high catalytic activity of methanol, excellent anti-toxic properties, and high electrochemical stability. The ECSA can be up to 87.41 m^2^/g at a scan rate of 50 mV/s. They also have the lowest oxidation potential of CO. These excellent electrochemical performances are attributed to the uniform dispersion of PtNiCo nanoparticles, good conductivity, stability, and large specific surface area of the rGO carrier. The synthesized PtNiCo/rGO nanoparticles have an average size of 17.03 ± 1.93 nm. We also investigated the effect of catalyst material size on electrocatalytic performance, and the results indicate that PtNiCo/rGO NC catalysts can replace anode catalyst materials in fuel cell applications in the future.

## 1. Introduction

Fuel cells have been considered green energy, renewable, and efficient energy devices in recent years due to environmental and energy challenges [1]. Direct methanol fuel cells (DMFCs) have attracted significant attention due to their high energy density and the abundance of liquid methanol [2,3]. The advantages of DMFCs are the simple structure of the system [4], low pollution [5], low operating temperature [6], and high energy conversion efficiency. They mainly convert methanol to produce electric energy through the catalyst of the electrode. Therefore, they can be used in small and portable electronic products such as laptops and mobile phones [7,8]. However, the electro-oxidation reaction of methanol is very complicated and slow, which is needed to improve the rate of electro-catalytic reaction. Moreover, the cost of DMFC systems is still very high, so the large-scale application of DMFCs is still quite limited [9].

In DMFC, methanol is oxidized to produce H^+^, e^−^, and CO_2_ according to the reaction formula CH_3_OH + H_2_O → CO_2_ + 6H^+^ + 6e^−^. However, carbon–oxygen bonds in methanol are not easy to break, so the methanol electro-oxidation reaction (MOR) on the anode is critical to the overall performance of DMFCs [10,11]. Pt-based alloys are widely used as highly reactive anode catalysts as an important component of DMFCs [12,13]. However, Pt-based alloys are easily hindered by the formation of toxic intermediates during electrochemical oxidation [12,14]. To avoid catalyst poisoning and oxygen reduction reaction (ORR), PtM alloys are proposed to have enhanced performance compared to pure platinum [13,15]. The electrocatalytic ability of Pt alloy increases because of the ligand effect and bifunctional effect [16]. It has been reported that Pt–Ni nanocrystals always have higher electrochemical activity, and Pt-Co nanocrystals are more stable than other Pt–M alloy materials attributed to the contribution of fine-tuned electronic structure [17]. Compared with monometallic or bimetallic catalysts, the ternary metallic catalysts have better electrochemical performance and significant catalytic activity. Therefore, adding other non-precious transition metals such as Ni, Co, Ru, etc. to replace the expensive Pt can reduce the consumption of expensive Pt materials while maintaining excellent performance [18]. Rethinasabapathy et al. [19] synthesized ternary PtRuFe nanoparticles supported by N-doped graphene as efficient methanol oxidation exhibiting higher ECSA. MOR activity is two to three times higher compared to other mono- and bimetallic catalysts. The addition of Fe significantly reduces the amount of Pt used in fuel cells. Other high-quality ternary PtRhCu nanocrystals with highly dendritic nanostructures were synthesized. The high specific activity and mass activity of the catalyst are due to the synergistic effect between Pt, Rh, and Cu elements and their highly dendritic nanostructure [20]. Sui et al. [21] prepared ternary Au@PdNi core–shell nanoparticles by a facile method. The results indicated that the electronic effects and the core–shell nanostructure played an important role in enhancing the catalytic activity and stability. They also enhanced the toxicity resistance of catalyst intermediates [22], improved the performance and durability of catalysts, and increased the overall energy conversion efficiency [23]. Pt_1-x-y_Ir_x_Ni_y_ nanocrystals were synthesized by a one-step process at room temperature and showed excellent tolerance to poisoning and stability [24]. In addition, ternary PtIrCu nanocrystals exhibit high durability and toxicity tolerance due to their large surface area, composition, and strain effects [25]. Lee et al. [26] developed a carbon-loaded PtRuNi/C ternary electrocatalyst. Compared to Pt/C and PtRu/C catalysts, PtRuNi/C catalysts exhibit enhanced CO tolerance. PtNiCo ternary alloy nanoframe crystals exhibit excellent activity and durability as efficient electrocatalysts for hydrogen evolution reaction (HER) [27]. The stable and highly efficient ordered Pt_2_CoNi ternary alloy electrocatalyst has 5–6 times higher electrocatalytic ORR activity than commercial Pt/C catalysts [28]. Therefore, the ternary alloy Pt-Ni-Co nanoparticles have higher catalytic activities, better stability, and a CO anti-poisoning effect [29].

Several methods to prepare electrocatalysis materials have been developed. Bhunia et al. [30] exhibited a simple one-pot and one-step solvothermal synthesis of PtAuNi nanoparticles as electrocatalysts with a diameter distribution of 3–7 nm by heating in an oven at 200 °C for 72 h. Sial et al. [31] used a typical hydrothermal method synthesis of trimetallic PtCoFe alloy nanosheets to obtain fuel cell catalysts with excellent electrocatalytic activity and durability. Lee et al. [26] reported that a PtRuNi/C ternary metal-based electrocatalyst can be possibly used as a CO-tolerant anode catalyst for PEMFC. In their study, a protective coating was used to prepare a product with a Pt-rich shell to prevent the Ni dissolution and sintering effect. Nugraha et al. [32] synthesized mesoporous AuCuNi alloy films by electrodeposition from an electrolyte solution containing three metal precursors with a micellar sacrificial template at the fixed applied potential. The mesoporous AuCuNi films were synthesized for nonenzymatic glucose sensing with high sensitivity, selectivity, and low detection limit. Yang et al. [13] fabricated porous Pt–Pd nanoparticles by a reflux method. Hong et al. [33] developed a galvanic replacement method to obtain Pd–Pt with Pd nanocrystals with different shapes as sacrificial templates. Wang, H. et al. [34] prepared a Pt nanocomposite by dopamine self-polymerization and a displacement reaction. Choi et al. [35] showed that extremely dispersed Pt and PtNi nanoparticles can be synthesized on supports by an impregnation process employing thiometallate precursors. Pd−Co nanowires with a jagged appearance were obtained via the template-confined electrodeposition first and afterward excessive etching in phosphoric acid by Wang, C. et al. [14]. Wang, P. et al. [36] fabricated PtPdCu porous nanodendrites and nanocubes by using a surfactant assisted coreduction method with a solvent of water. However, the methods described above have several problems, including a comparatively low price–performance ratio, more elementary reaction steps, and inefficient synthesis. Therefore, more economical and novel tactics need to be utilized in the synthesis of electrocatalysts to improve catalytic properties. In this study, we found a simple, efficient, one-step synthesis, time-saving, environmentally friendly, non-metallic protective coating, and template-free method for the preparation of electrocatalysts.

Microwave irradiation indicates ultra-high frequency electromagnetic waves with a certain wavelength. The range is from 1 m to 1 mm and the frequency range of 300 MHz–300 GHz [37]. The advantages of using microwaves compared to traditional heating methods are uniform heating, high speed [38], and energy efficiency. On the other hand, the heating material does not need to be in direct contact with the heat source, so the thermal resistance effect in the heat transfer process can be reduced. Microwave-assisted heating is a simple, effective, and energy-efficient heating method that has been successfully applied in organic synthesis [39], functionalization of carbon nanotubes [40], and preparation of exfoliated graphite [41]. Pipus et al. [42] found that the esterification reaction of benzoic acid is a slow process and requires several days to reach equilibrium at 80 °C. However, microwave heating (140 °C, 7 atm) was able to increase the rate of the esterification reaction in a short time. Yangá Lee et al. [43] showed that the nanoparticles obtained by microwave-assisted heating with the smallest particle size could be uniformly dispersed on the carbon carrier and had high electrocatalytic activity.

In this study, a ternary metallic nanocatalyst was synthesized by the microwave-assisted method. In addition, the appropriate carbon support is also essential for the conduction of electrons and the dispersion of precious metal particles during the catalyst design process. Graphene is chosen as a carbon carrier due to its unique advantages such as large specific surface area, flexible two-dimensional structure, high mechanical property [44], and good conductivity [17]. The loading of PtNiCo nanoparticles on graphene improves the utilization of the metal and its uniform dispersion on the graphene. It contributes to the accessibility of surface active sites and electron transfer kinetics [23]. The PtNiCo/rGO nanocomposite catalysts were prepared by GO with different ratios of Pt, Ni, Co precursors in an ethylene glycol solution microwave-assisted system at different temperatures for 20 min. In this process, a reductant is used to obtain better performance to eliminate oxygen-containing functional groups [17]. The PtNiCo particles were successfully loaded on the supports and the structural characteristics of the prepared catalysts were evaluated by transmission electron microscopy (TEM), energy-dispersive X-ray spectroscopy (EDX), X-ray diffraction (XRD), and Raman. The electrochemical measurements included cyclic voltammetry (CV) scanning, CO stripping, and chronoamperometry (CA).

## 2. Materials and Methods

### 2.1. Materials

Graphite powder (99.99%) and potassium hexachloroplatinate (K_2_PtCl_6_, 99.99%) were purchased from Alfa Aesar (Haverhill, MA, USA). Sodium nitrate (NaNO_3_, 99.5%), potassium permanganate (KMnO_4_, 99.3%), and nickel chloride hexahydrate (NiCl_2_·6H_2_O, 96%) were purchased from Hayashi Pure Chemical (Osaka, Japan) and cobalt(II) nitrate hexahydrate (Co(NO_3_)_2_·6H_2_O, 99%) was purchased from JT Baker Chemicals (Phillipsburg, NJ, USA). Hydrogen peroxide (H_2_O_2_, 30%) was purchased from Showa Chemical (Tokyo, Japan). Ethylene glycol (EG, 99.9%) was purchased from TEDIA (Fairfield, OH, USA). Hydrochloric acid (HCl, >35%) and potassium hydroxide (KOH, 85%) were purchased from Union Chemical Works (Hsinchu, Taiwan). Liquid Nafion (5 wt%) was purchased from DuPont (Wilmington, DE, USA). Sulfuric acid (H_2_SO_4_, 97%), acetone ((CH_3_)_2_CO, 95%), and methanol (CH_3_OH, 99.5%) were purchased from Nihon Shiyaku Reagent (Kyoto, Japan). Millipore water (18 MΩ) was used for all electrochemistry measurements. All chemicals used in this experiment were analysis reagents (A.R.).

### 2.2. Fabrication of Graphene Oxide by a Modified Hummers Method

GO was prepared by the modified Hummers method from graphite powder [45]. Seventy milliliters of H_2_SO_4_ was added to an ice bath and cooled to 5 °C. Graphite powder, NaNO_3_, and KMnO_4_ were added to the flask and stirred well for 2 h. Then, 300 mL of deionized water was added and the color of the solution changed to yellowish brown, then 10 mL of 30% hydrogen peroxide was added to stop the reaction. After suction and filtration, the sample was put into 500 mL of 5% hydrochloric acid to remove the metal ions, then washed repeatedly with DI water until neutral. The products were then dried overnight in an oven at 80 °C to obtain graphene oxide.

### 2.3. Preparation of Samples

**Figure 1:**
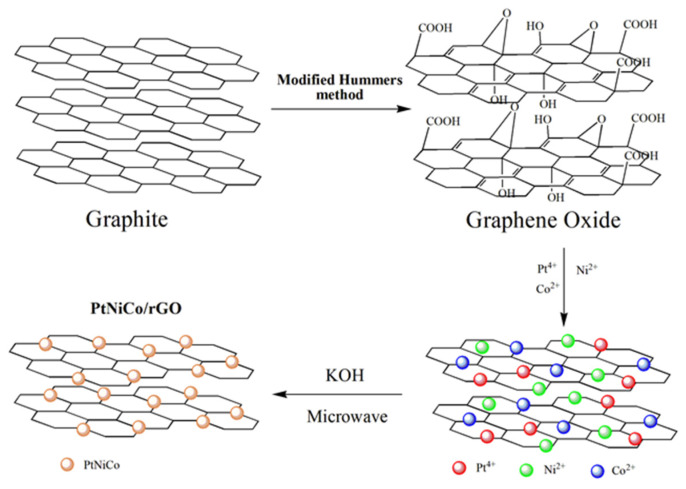
Illustration of the formation of the PtNiCo/rGO.

A 0.02 M K_2_PtCl_6_, 0.02 M NiCl_2_, 0.02 M Co(NO_3_)_2_ solution was mixed with 30 mL of ethylene glycol, 20 mg GO, and stirred for 30 min. The resulting homogeneous dark brown solution was adjusted to pH 10 with 0.2M KOH solution and transferred to 100 mL Teflon digestion vessels. The solution was heated at 200 °C for 20 min by a microwave system and then cooled to room temperature naturally. The samples were filtered and dried in an oven at 70 °C. Nanocomposites at different temperatures were prepared using a similar process. PtNiCo/rGO was synthesized at 160, 180, 200, and 220 °C, labeled as PtNiCo/rGO 160, PtNiCo/rGO 180, PtNiCo/rGO 200, and PtNiCo/rGO 220, respectively. PtNi_2_Co/rGO, PtNiCo/rGO, and PtNiCo_2_/rGO were synthesized in the mole ratios of Pt:Ni:Co 1:2:1, 1:1:1, and 1:1:2, respectively. A graph of the synthesis process is shown in Figure 1.

### 2.4. Preparation of Catalyst Ink

The PtNiCo/rGO ink was prepared by adding 4 mg of sample powder to 0.8 mL of DI water and 0.2 mL of ethanol solution. The platinum working electrode was polished and used as a working electrode. It was coated with 15 μL of the above catalyst ink and 15 μL of Nafion ionomer, and dried at room temperature. After the electrodes were dried, electrochemical measurements could be carried out.

### 2.5. Structural Catalyst Characterization

PtNiCo/rGO nanocomposite was synthesized by a microwave (Flexiwave T660, Milestone srl, Sorisole, Italy). The crystal structure of the samples was measured by X-ray diffraction (XRD, D8A25 eco, BRUKER Co. Ltd., Billerica, MA, USA) with CuKα X-ray radiation (λ = 1.5418 Å) at 40 kV and 25 mA. The morphology of the samples was observed by transmission electron microscopy (TEM, Hitachi H-7500, Tokyo, Japan) with an accelerating voltage of 80 kV. The elemental composition of the prepared nanocomposites was analyzed by energy dispersive X-ray analysis (EDS, INCA x-act) equipped with scanning electron microscopy (SEM, JEOL JSM-6390, Tokyo, Japan). Raman spectra were analyzed with a nitrogen-cooled CCD detector (Shamrock 750 spectrograph, Andor Technology Ltd., Belfast, Northern Ireland, UK). A randomly polarized 533 nm laser with an excitation power of 0.45 mW was used.

### 2.6. Electrochemical Characterization

The electrochemical measurements were carried out on a Bio-Logic CLB-500 electrochemical Workstation (Knoxville, TN, USA) at room temperature. The electrochemical test was carried out with a three-electrode apparatus: a platinum working electrode (diameter: 2 mm), a Pt wire, and Ag/AgCl electrodes as the working, counter, and reference electrodes, respectively. The measured potentials are all compared with Ag/AgCl electrodes for convenience of comparison. Cyclic voltammetry (CV), CO stripping, and chronoamperometry (CA) were employed to estimate the electrochemical activity and stability of catalysts. CV and CA techniques were employed to measure at room temperature and in a N_2_ saturated environment, while CO stripping is tested under saturated CO gas. The ECSA was calculated from the hydrogen desorption peak of the CV method in 0.5 M H_2_SO_4_ electrolyte, which was conducted by cycling the potential between −0.25 and 1.0 V, with a scan rate of 50 mV/s. CO stripping evaluates the ability of the catalyst to metabolize toxic substances, and its scanning potential is between −0.2 and 1.0 V, carried out in 0.5 M H_2_SO_4_ electrolyte. Electrochemical activity and catalytic ability of the catalysts for MOR were determined by CV and CA methods in 0.5 M H_2_SO_4_ + 1.0 M CH_3_OH electrolytic solution.

## 3. Results and Discussion

### 3.1. Characterization of PtNiCo/rGO

#### 3.1.1. XRD Analysis

**Figure 2:**
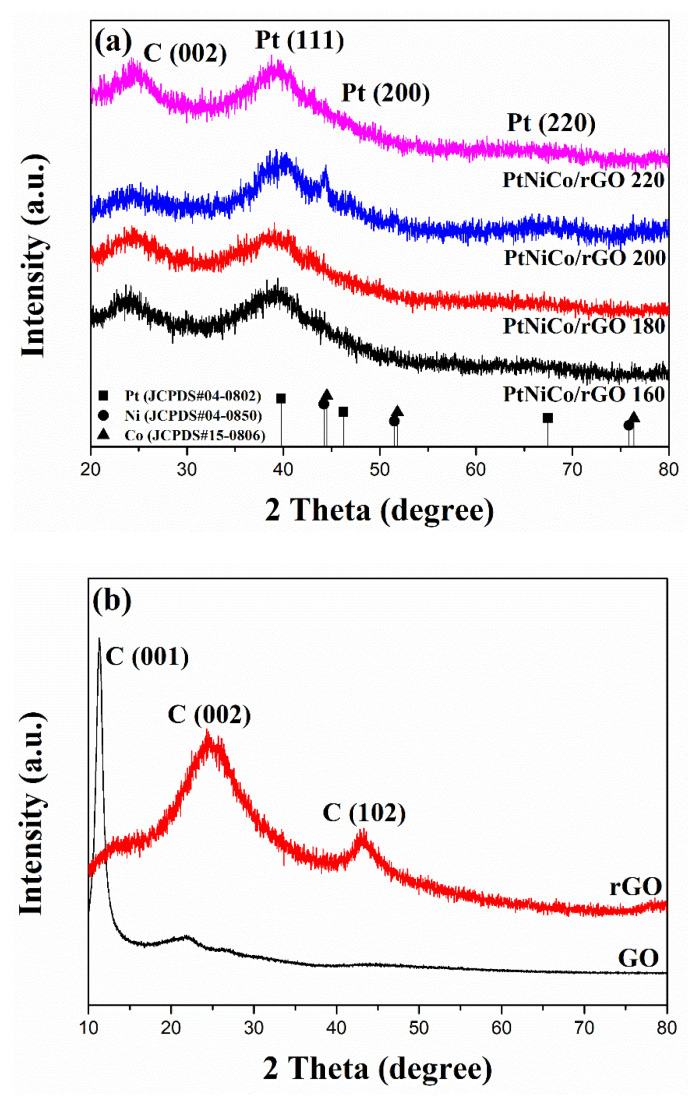
XRD patterns of (**a**) PtNiCo/rGO of different temperatures. (**b**) GO and rGO.

The crystal structure of nanocomposites was analyzed by powder XRD. The diffraction peaks of these Pt-rich phases are fase center cubic (fcc) structures (Figure 2a) [17]. Note that the peaks become progressively broader after thermal treatment. The diffraction peaks are located at the corresponding angles of 39.7°, 46.2°, and 67.4°, and the lattice constants are (111), (200), and (220) diffraction lattice planes, respectively. The diffraction peaks of Ni and Co are located at the top of the diffraction peak, corresponding to the angles of 44.5°, 51.8°, 76.4° and 43.7°, 51.0°, 74.7°, respectively. Based on the Joint Committee on Powder Diffraction Standards (JCPDS) card number #04-0802, it is determined that Pt metal represents Pt (111), Pt (200), Pt (220) crystallographic planes. In the PtNiCo/rGO 160, PtNiCo/rGO 180, PtNiCo/rGO 200, and PtNiCo/rGO 220 samples, we found a much broader peak at 2θ = 26°, while the diffraction peak at 2θ = 11.9° disappeared significantly. This indicates that GO has been reduced to rGO (Figure 2b). The diffraction peak at 26° can be marked as rGO, with no additional peaks for phase separation structures such as pure Ni or Co. This indicates an excellent degree of alloying between Pt, Ni, and Co [46]. The XRD results showed that the diffraction peaks of Ni and Co in composites are not obvious and may be related to the small amount of Ni and Co in composites [47,48,49]. However, the presence of Ni and Co can also be explained by a slight shift of the Pt (111) peak to a higher angle in the XRD analysis. The peak position of Pt (111) shifts to higher 2θ values due to the introduction of smaller Ni and Co atoms, resulting in reduced lattice distances and a composite with Pt to form the PtNiCo ternary alloy [28,50,51]. The catalysts exhibited broader shoulder peaks, assigned to the characteristic peak of the rGO support [52]. Moreover, the higher diffraction shifts of Pt (111) of the synthesized nanocomposites at 200 °C indicate that the synthesis is relatively complete and PtNiCo ternary alloy is better.

#### 3.1.2. Morphological Characterization

**Figure 3:**
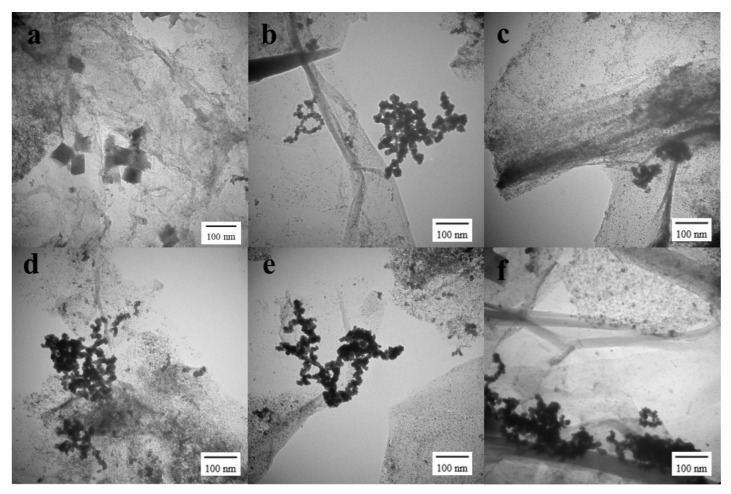
Morphology and structural characterization. TEM images of (**a**) PtNi_2_Co/rGO 200, (**b**) PtNiCo/rGO 200, (**c**) PtNiCo_2_/rGO 200, (**d**) PtNiCo/rGO 160, (**e**) PtNiCo/rGO 180, (**f**) PtNiCo/rGO 220.

**Figure 4:**
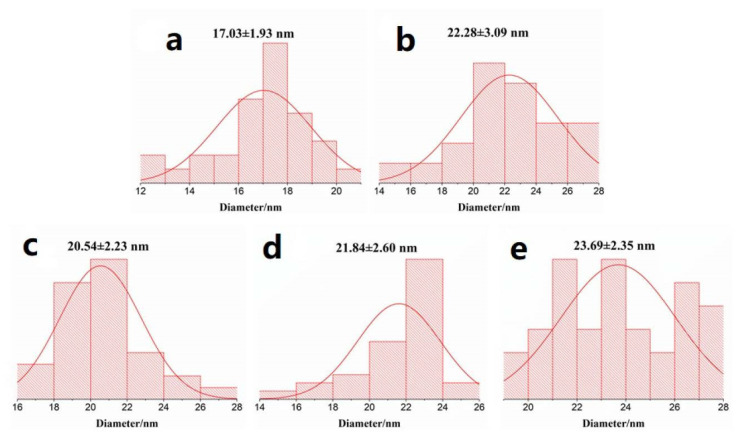
Particle size distribution of (**a**) PtNiCo/rGO 200, (**b**) PtNiCo_2_/rGO 200, (**c**) PtNiCo/rGO 160, (**d**) PtNiCo/rGO 180, (**e**) PtNiCo/rGO 220.

Figure 3a–c show the synthesized PtNiCo/rGO ternary alloys with different ratios. The morphology of the ternary alloy/reduced graphene oxide nanocomposite was analyzed by TEM. Moreover, the dispersion of the catalyst was observed to find the optimum ratio of the atomic catalyst. In these samples, the TEM diagrams display two-dimensional images of PtNiCo/rGO 200 and PtNiCo_2_/rGO 200 with a characteristic size of about 17.03 ± 1.93 nm and 22.28 ± 3.09 nm (Figure 4a,b). The ternary alloy PtNiCo/rGO 200 has an average particulate size of less than 20 nanometers.

Furthermore, PtNiCo/rGO in Figure 3 shows a large number of PtNiCo nanoparticles surrounded by the rGO nanosheets. The metal precursor solutions were reduced to PtNiCo ternary alloy nanoparticles with ethylene glycol at different microwave temperatures, which were dispersed on the surface or embedded in the layered structure of rGO, as shown in Figure 3d–f. The 2D sheet-like structure and slight folds can be observed in TEM images. Figure 4 shows the particle size and distribution of various samples and various reaction conditions. The particles formed gradually at 160 °C and 180 °C, but the reaction showed that the metal ions did not fully composite into the ternary metallic nanoparticles. The particles have a mean radius of about 21 nm and a wide distribution width. The particle size decreases as the temperature rises to 200 °C. This is due to the larger number of seeds growing and the particles reacting more thoroughly. The metal ions can be converted to ternary metallic nanoparticles and exhibit an average radius of 17.03 ± 1.93 nm and a narrower distribution width. Larger particles are formed as the temperature increases (Figure 4a,b). At this time, the agglomeration mechanism leads to more clusters agglomerating at higher temperatures and forming nanoparticles [53]. The results show that the average particle size of the synthesized nanoparticles at 200 °C is the smallest, 17 nm, and has a relatively dispersed structure compared with other synthesis conditions. The main reason for the small particle size is the short time and fast rate of nucleation at optimal temperature conditions [54,55]. Under other synthesis conditions, the average particle size was about 20 nm (Figure 4). The temperature-dependent agglomeration is the dominant mechanism of the particle size difference [56,57]. The results showed that the dispersed PtNiCo nanoparticles were easily grown on rGO and were active on electrochemical properties [58].

**Figure 5:**
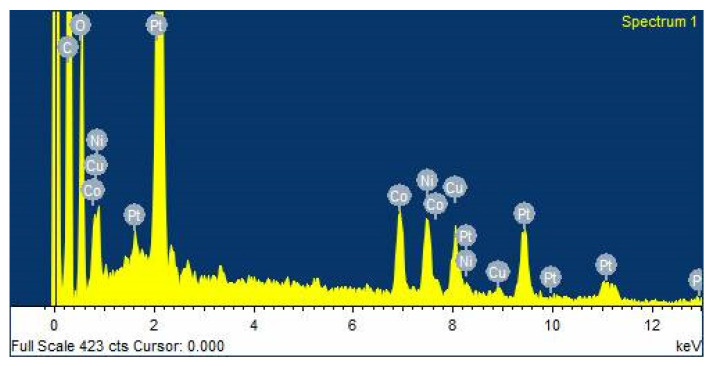
EDX spectrum of PtNiCo/rGO 200 composite.

The composition of the synthesized nanocomposites was obtained by energy dispersive X-ray analysis (EDS) equipped with SEM for evaluation. The EDX spectrum of PtNiCo/rGO 200 in Figure 5 shows that it consists mainly of three metal elements, Pt, Ni, and Co, in the sample. The peaks of Ni and Co can be clearly detected in the EDX image, and the atomic percentages of Pt, Ni, and Co are 2.73, 2.67, and 2.69%, respectively. The atomic composition of the composite is almost identical to that of the metal precursor solution. The EDX image demonstrates the presence of C elements in rGO, while O elements are mainly derived from residual oxygen-containing functional groups in rGO. Other elements can also be observed in the figure, mainly added from the material during the experiments. The appearance of Cu peaks originates from the copper gels used in the analysis [59]. The EDX images provide evidence for the presence of Pt, Ni, Co, rGO, and PtNiCo atomic ratios in the electrocatalyst.

#### 3.1.3. Raman Spectrum

**Figure 6:**
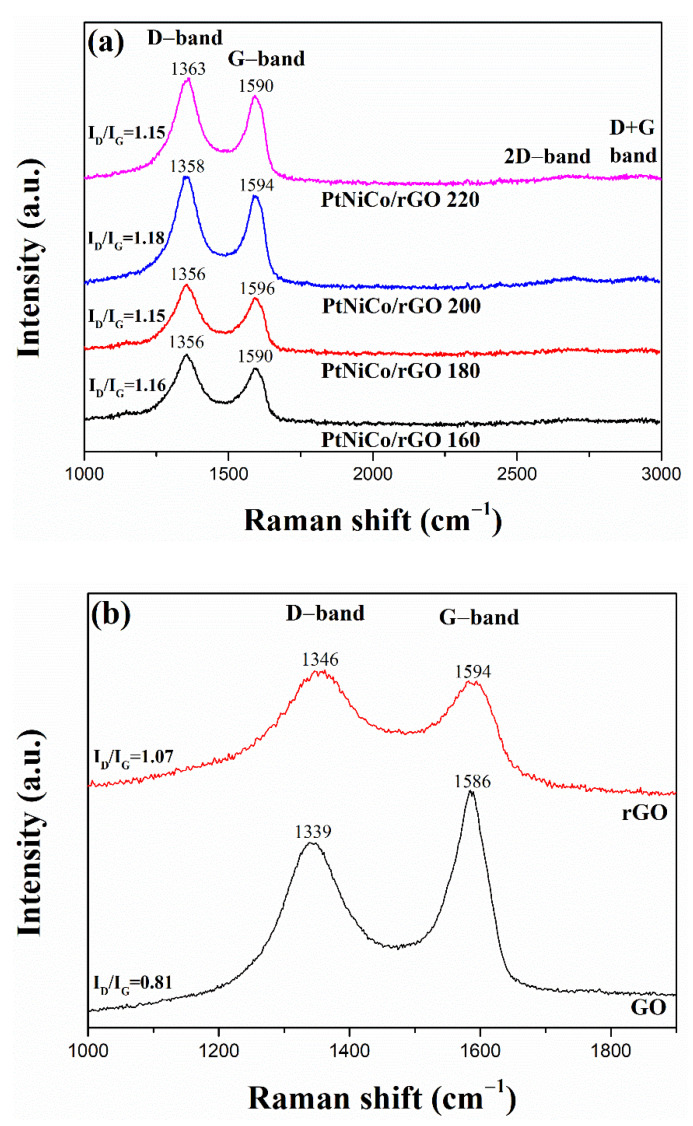
Raman spectra of (**a**) PtNiCo/rGO at various temperatures. (**b**) GO and rGO.

Figure 6a shows the Raman spectra of the samples in the range of 1000–3000 cm^−1^. They all display obvious D bands and G bands originating from carbon sp^2^ domains and structural defects [60]. The G band is attributed to the E_2g_ mode of C sp^2^ atoms and the D band arises due to the A_1g_ symmetry [61]. The structural disorder of a graphitic structure could be estimated according to the I_D_/I_G_. It can be seen from Figure 6a,b that the D-band and G-band of GO, rGO, PtNiCo/rGO 160, PtNiCo/rGO 180, PtNiCo/rGO 200, and PtNiCo/rGO 220 were 1339 cm^−1^ and 1586 cm^−1^, 1346 cm^−1^ and 1594 cm^−1^, 1356 cm^−1^ and 1590 cm^−1^, 1356 cm^−1^ and 1596 cm^−1^, 1358 cm^−1^ and 1594 cm^−1^, and 1363 cm^−1^ and 1590 cm^−1^, respectively. The I_D_/I_G_ of GO, rGO, PtNiCo/rGO 160, PtNiCo/rGO 180, PtNiCo/rGO 200, and PtNiCo/rGO 220 were 0.81, 1.07, 1.16, 1.15, 1.18, and 1.15, respectively. Afterwards, GO was reduced to rGO because large numbers of sp^3^ carbon were reduced to sp^2^ carbon, which increases the I_D_/I_G_ value. In general, the D-band and G-band intensities were reduced after the PtNiCo nanoparticles were composited with reduced graphene oxide. This is because the exposred area of the rGO sheet to the excitation light in PtNiCo/rGO nanocomposite is reduced in Raman measurements. The broadening of the D-band and G-band of the nanocomposite is caused by the lattice strain between rGO and PtNiCo [62].

The I_D_/I_G_ value of PtNiCo/rGO is higher than that of GO, indicating that the double bond of GO is broken and composited with metal nanoparticles. The larger the I_D_/I_G_ value, the more successfully the sample is composited [19]. In addition, the I_D_/I_G_ ratio could be related to the existence of defects in graphene structure as a result of electronic interaction with PtNiCo metal nanoparticles, affirming the reduction of the functional groups during microwave-assisted treatment. These defects introduced by GO act as the anchoring sites for the attachment of PtNiCo metal nanoparticles. According to the description in the literature, the increase in the intensity is related to the PtNiCo nanoparticles incorporated into the rGO as both peaks increased similarly in intensity [63]. A higher degree of graphitization is beneficial to promote the overall conductivity of the final product and enhance the electrochemical activity. In addition, the crystallinity of the 2D band (about 2700 cm^−1^) in PtNiCo/rGO 200 is higher, which is predicted to be more corrosion resistant in DMFC [61]. The wide 2D band shows the multilayer structure of rGO, affirming the existence of graphene and mainly coming from a double resonance process that links phonons to the electronic band structure [64]. The 2D and D + G band peaks, near 2700 and 2900 cm^−1^, correspond to the combination mode induced by the disorder.

### 3.2. Electrochemical Measurements

#### 3.2.1. CV and Mass Activity Analysis

**Figure 7:**
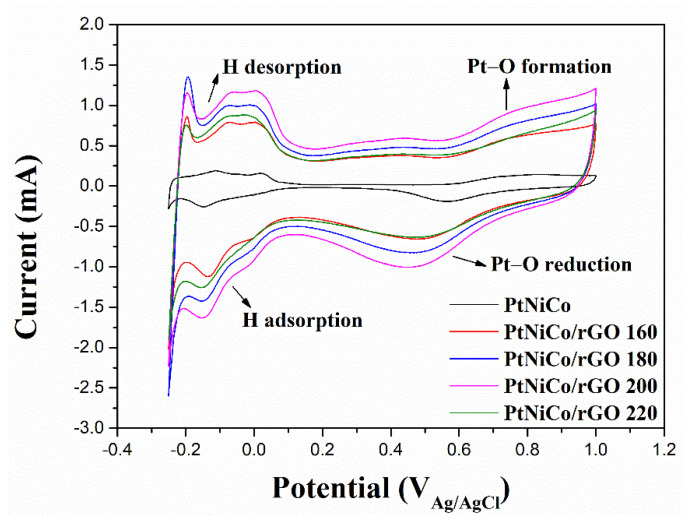
Electrocatalytic performance of PtNiCo, PtNiCo/rGO 160, PtNiCo/rGO 180, PtNiCo/rGO 200, and PtNiCo/rGO 220 catalysts. Cyclic voltammograms obtained at room temperature in N_2_-purged 0.5 M H_2_SO_4_ aqueous solution at scan rate of 50 mV/s.

To obtain the ECSA of the catalysts, they were prepared as a slurry and coated on the Pt working electrode. In this study, CV was used to measure and analyze the characteristics of the catalysts. Figure 7 shows the CV curves of the different catalysts recorded in N_2_-purged sulfuric acid solution at a scan rate of 50 mV/s in the potential range of −0.2 V to 1.0 V. CV curves are divided into three parts to show the typical Pt-H under the potential deposition region, double-layer region, and Pt oxide region. Typical absorption and desorption of hydrogen occur in the low potential region. There is a significant redox peak from −0.2~0.1 V, indicating the adsorption/desorption of hydrogen on Pt [65].

The corresponding ECSA was calculated by integrating the hydrogen desorption zones and all the CV curves were normalized. According to the following formula [66]:

$$\mathrm{ECSA}\;\left({\mathrm{cm}}^{2}g^{-1}\right)=\frac{\mathrm{charge}\;\left(Q_{H},\;\mu C\;{\mathrm{cm}}^{-2}\right)}{210\;\left(\mu C\;{\mathrm{cm}}^{-2}\right)\;\times \;\mathrm{electrode}\mathrm{loading}\;\left(g\mathrm{Pt}{\mathrm{cm}}^{-2}\right)}$$

where *Q*_H_ (μC cm^−2^) is the hydrogen desorption charge, 210 (μC cm^−2^) is the charge required to oxidize the layer of hydrogen on Pt, and the electrode loading (g Pt cm^−2^) is the Pt working electrode loading. The individual ECSAs of these catalysts were measured as 18.19, 61.43, 82.65, 87.41, and 41.41 m^2^/g for PtNiCo, PtNiCo/rGO 160, PtNiCo/rGO 180, PtNiCo/rGO 200, and PtNiCo/rGO 220. The PtNiCo/rGO 200 ECSA is larger than the others because of the dispersed and well-anchored Pt nanoparticles on the surface of rGO nanocomposites. The nanocomposite of 200 °C has a good distribution of fine PtNiCo nanoparticles, which is attributed to the largest ECSA. This result can be seen from the TEM image, which has the smallest particle size distribution under the 200 °C condition.

**Figure 8:**
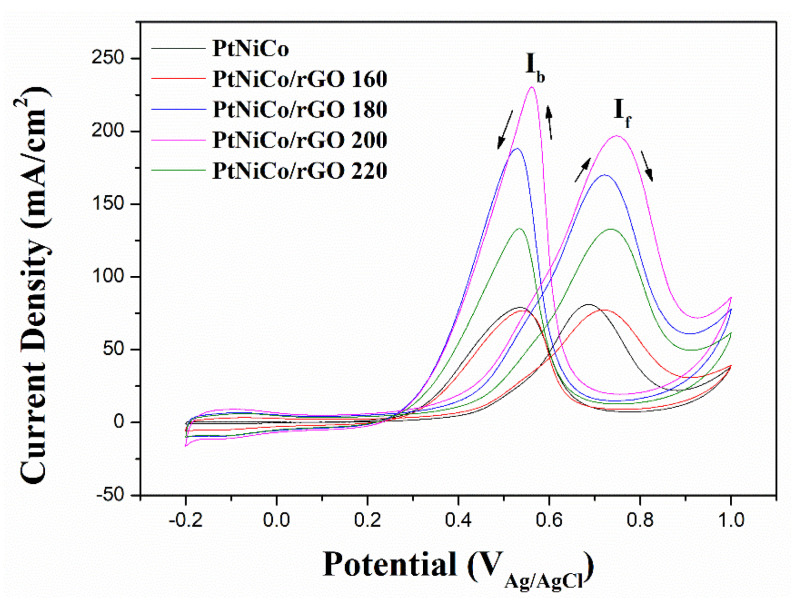
CV curves of methanol oxidation on PtNi_2_Co/rGO, PtNiCo/rGO, PtNiCo, and PtNiCo_2_/rGO in N_2_-saturated 0.5 M H_2_SO_4_ + 1.0 M CH_3_OH solution, sweep rate 20 mV/s.

**Figure 9:**
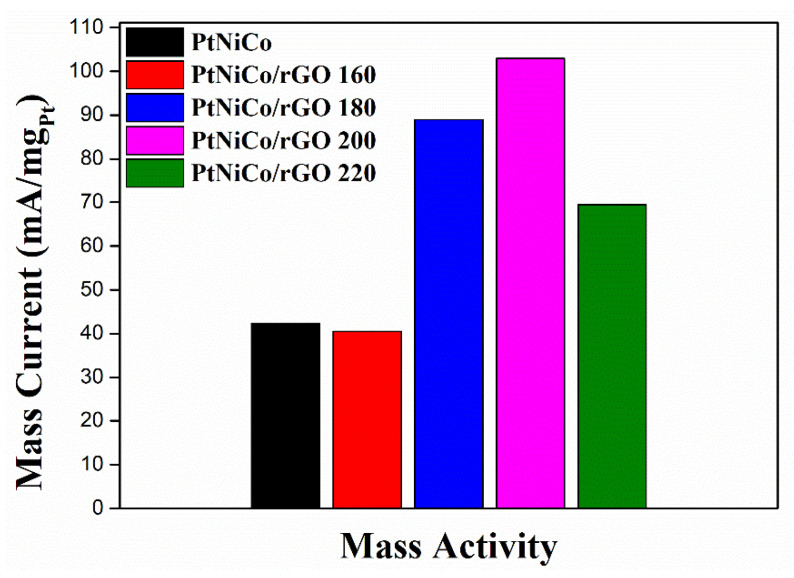
Histogram of mass activities of different catalysts for MOR.

To investigate the different ratio catalysts in terms of the catalytic activity of methanol oxidation, CV measurements were performed (Figure 8). In the forward scan, the positive scan anode peak is about 0.7 V compared to Ag/AgCl, which is caused by the oxidation of methanol. In the reverse scan, the oxidation peak appears at about 0.5 V, possibly owing to incompletely oxidized carbonic matters formed in the forward scan. The current density of CVs at ∼0.7 V on PtNiCo/rGO 200 is 196.82 mA cm^−2^, which is about 2.5 times larger than the PtNiCo (80.89 mA cm^−2^). To clearly illustrate the catalytic capacity, MOR performance is compared by normalizing peak currents to specific areas and qualities of Pt expressed as mass activity [67]. The mass activity of all catalysts is shown in Figure 9. It can be observed that PtNiCo/rGO 200 has the highest mass activity (102.96 mA mg^−1^) compared with other catalysts, indicating that the PtNiCo/rGO 200 catalysts had the highest catalytic activity for MOR.

The results show that PtNiCo/rGO 200 has the highest catalytic activity and mass activity in these synthesized catalysts. The high performance of PtNiCo/rGO is attributed to the electronic state of Pt, which is mainly because the crystal structure and electronic structure of Pt nanoparticles can be changed by introducing other metals, thus improving the binding energy between Pt and toxic species [9,68]. In addition, the TEM images of PtNiCo/rGO 200 can significantly improve the catalytic activity due to the dispersion of small PtNiCo nanoparticles on rGO [59]. This is attributed to the fact that the presence of Ni and Co can mitigate Pt poisoning, resulting in the higher electrochemical activity of PtNiCo/rGO [69,70]. From the Raman spectrum, PtNiCo/rGO 200 has the highest I_D_/I_G_ intensity, meaning that a greater degree of composite PtNiCo and rGO, which will also enhance the electrocatalytic activity [71].

#### 3.2.2. CO Stripping Measurements

**Figure 10:**
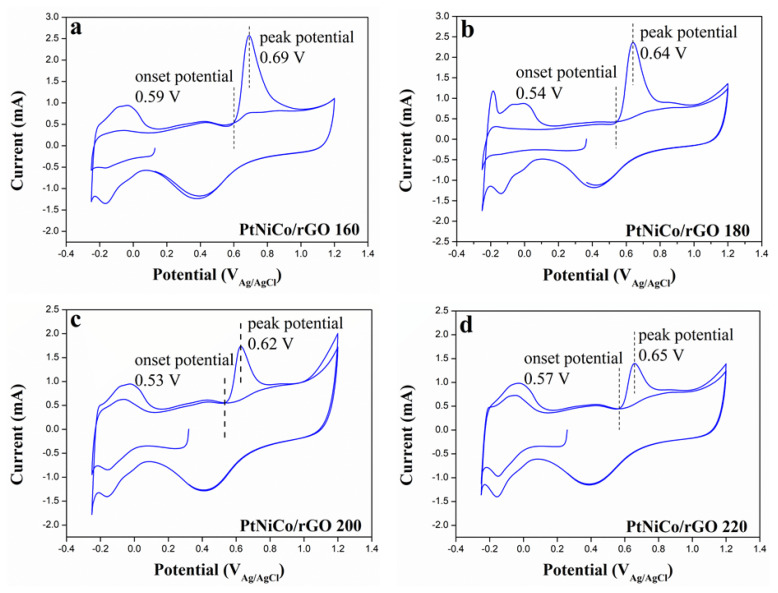
CO stripping curves of (**a**) PtNiCo/rGO 160, (**b**) PtNiCo/rGO 180, (**c**) PtNiCo/rGO 200, and (**d**) PtNiCo/rGO 220 in CO−purged 0.5 M H_2_SO_4_ solution at scan rate of 50 mV/s.

CO stripping experiments were carried out on the oxidative removal of CO. The CO gas is pumped to make the CO adhere to the catalyst at a lower potential, and then the CV test is carried out. Figure 10 shows the voltammograms of CO oxidation for various samples at various peak potentials. In addition, the CO oxidation potentials (vs. Ag/AgCl) of PtNiCo/rGO 160, PtNiCo/rGO 180, PtNiCo/rGO 200, and PtNiCo/rGO 220 were 0.69 V, 0.64 V, 0.62 V, and 0.65 V vs. Ag/AgCl, respectively. These peaks disappear after the first scan in the forward direction. The PtNiCo/rGO 200 expresses a more negative peak potential and onset potential, indicating that the affinity between Pt and CO is weakened [72]. The results show that the introduction of Ni and Co elements can improve the oxidation ability of CO [73]. The electronic interaction between Pt, Ni, and Co could lead to the removal of CO poisoning substances and enhance the stability of the electrodes. Therefore, there are fewer intermediates adsorbed on the surface of PtNiCo/rGO 200, and the oxidation efficiency of CO_ads_ is higher, which is beneficial to increase catalytic active sites [73].

The synergistic effect of CO electrooxidation can be clearly seen from the oxidation onset potential to more negative values. Due to the catalytic effect, PtNiCo/rGO 200 could activate CO at lower potentials than the other catalysts. This result also contributes to illustrating the higher activity of PtNiCo/rGO for the oxidation of methanol at 200 °C [74].

Table 1 shows the comparison of the ECSA, forward peak current density for methanol oxidation, and CO oxidation potential of PtNiCo/rGO 200 to those of various electrocatalysts investigated in previous studies. The ECSA and methanol oxidation current density of PtNiCo/rGO 200 were higher than those of the other materials previously studied. These results indicated the enhanced Pt hydrogen absorption/desorption area and methanol electrocatalytic activity of PtNiCo/rGO 200. In addition, the CO oxidation potential of PtNiCo/rGO 200 was mostly lower than other materials, showing better CO anti-poisoning ability.

#### 3.2.3. Chronoamperometric Study

**Figure 11:**
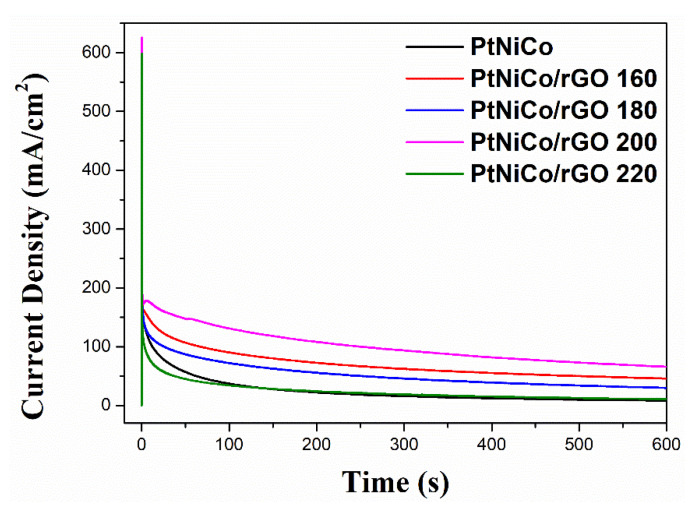
Chronoamperometric curves of PtNiCo, PtNiCo/rGO 160, PtNiCo/rGO 180, PtNiCo/rGO 200, and PtNiCo/rGO 220 in 0.5 M H_2_SO_4_ + 1.0 M CH_3_OH solution at constant potential of 0.70 V.

The catalytic stability of the catalyst for MOR in acidic media was further investigated by CA. Figure 11 shows the CA curve for the variation in current density with time. As the experiment progresses, the current decay of these catalysts slows down and gradually achieves quasi-steady state [75]. This is due to the formation of oxidation intermediate species such as CO, CH_3_OH, and CHO [76]. This causes the oxidation of methanol to produce adsorption on the Pt surface to hinder the active site. After testing at t = 600 s, the current density of PtNiCo/rGO 200 (65.92 mA/cm^2^) is still higher than that of PtNiCo (8.28 mA/cm^2^), PtNiCo/rGO 160 (45.86 mA/cm^2^), PtNiCo/rGO 180 (29.94 mA/cm^2^) and PtNiCo/rGO 220 (10.83 mA/cm^2^). The results show that PtNiCo/rGO 200 retains the highest steady current density and the highest initial current density when compared to the others. The catalysts supported on rGO show better stability than PtNiCo. This is due to the graphene sheets providing a number of oxygen groups to strengthen the interaction with Pt nanoparticles. As previously indicated, the existence of residual functional groups on reduced graphene oxide also contributes to the catalytic activity [77]. These results confirm that PtNiCo/rGO 200 shows great stability and higher intermediates of poison tolerance as a superior catalyst for MOR in DMFCs.

## 4. Conclusions

In summary, a ternary alloy structured was prepared with one-step microwave-assisted reduction for uniformly dispersed Pt-Ni-Co nanoparticles supported on rGO. The PtNiCo/rGO exhibited the highest electrochemical properties and the smallest average grain diameter (17 nm) at a microwave reaction temperature of 200 °C. The aggregation of PtNiCo is maximally suppressed by the rGO, attributed to the unique two-dimensional flexible microstructure of rGO. In addition, PtNiCo/rGO 200 had the highest I_D_/I_G_ values, indicating an increase in reduction. The PtNiCo/rGO nanocomposite presents excellent electrocatalytic ability in terms of high electrocatalytic activity, high poison tolerance, enhanced stability toward MOR compared with PtNiCo. Electrochemical measurements show that PtNiCo/rGO 200 nanoalloy displays functionality enhancement in both mass and catalytic activities over two times that of the pure PtNiCo catalyst. The unique dispersion of rGO and the synergistic effect between Pt, Ni, and Co improve the catalytic performance of PtNiCo/rGO composites. To satisfy the challenges of rapid fabrication and low environmental impact, we obtained PtNiCo/rGO using a rapid synthesis method with a simple process and low-cost precursors. The PtNiCo/rGO electrocatalysts have the potential to be used as catalysts with high electrocatalytic activity, CO resistance, and stability in DMFC. It provides a fast and reduced energy consumption fabrication for designing other high-performance catalysts, which is a great prospect in the application of fuel cell catalyst materials for the future.

## Figures and Tables

**Table 1:** ECSA and current density of methanol oxidation of various electrocatalysts in previous works.

Electrocatalyst	ECSA (m^2^/g)	Current Density (mA cm^−2^)	CO Stripping (V)	References
PtNiCo/rGO 200	87.41	196.82	0.62 vs. Ag/AgCl	This work
Pt/BG	58.8	~1.7	~0.8 vs. RHE	[4]
Pt-Ni/CNF 1:2	-	~2	~0.7 vs. RHE	[10]
Pt-Pd (9:1)	31.59	0.67	-	[13]
Pt_3_Pd_1_-CeO_2_/C	30.33	~4	~1.0 vs. RHE	[15]
Hollow Pt-Ni-Co NDs	57.0	3.8	~0.5 vs. SCE	[17]
PtCoFe	62.9	4.75	-	[23]
Au_41_Cu_46_Ni_13_	45.8	3.8	-	[25]
PtRuFe/rGO	56.4	1.33	-	[45]
Pd_59_Fe_27_Pt_14_ NMs	-	4.36	-	[50]

## Data Availability

All the data are available within the manuscript.
